# Maternal Health-Seeking Behavior: The Role of Financing and Organization of Health Services in Ghana

**DOI:** 10.5539/gjhs.v5n5p67

**Published:** 2013-05-30

**Authors:** Emmanuel Aboagye, Otuo Serebour Agyemang

**Affiliations:** 1 Department of Health Economics, Management and Policy, University of Oslo, Norway; 2 Department of Economics and Management, University of Ferrara, Ferrara, Italy

**Keywords:** health system, financing, organization, maternal health-seeking behavior, maternal outcomes, social structures, national health insurance scheme

## Abstract

This paper examines how organization and financing of maternal health services influence health-seeking behavior in Bosomtwe district, Ghana. It contributes in furthering the discussions on maternal health-seeking behavior and health outcomes from a health system perspective in sub-Saharan Africa. From a health system standpoint, the paper first presents the resources, organization and financing of maternal health service in Ghana, and later uses case study examples to explain how Ghana's health system has shaped maternal health-seeking behavior of women in the district. The paper employs a qualitative case study technique to build a complex and holistic picture, and report detailed views of the women in their natural setting. A purposeful sampling technique is applied to select 16 women in the district for this study. Through face-to-face interviews and group discussions with the selected women, comprehensive and in-depth information on health- seeking behavior and health outcomes are elicited for the analysis. The study highlights that characteristics embedded in decentralization and provision of free maternal health care influence health-seeking behavior. Particularly, the use of antenatal care has increased after the delivery exemption policy in Ghana. Interestingly, the study also reveals certain social structures, which influence women's attitude towards their decisions and choices of health facilities.

## 1. Introduction

### 1.1 Introduction to the Problem

The reduction of maternal mortality and morbidity recently is viewed as top priority area in many developing countries (Bergstrom, 1994). Recent studies point to the declining maternal mortality mostly high for countries in the developing and transition world (Okiwelu et al., 2007). However, since it is difficult to measure how much progress is occurring, estimates are usually assumed (Graham et al., 2001). Maternal mortality ratio in sub-Saharan Africa alone has reduced by only 1.6% per annum since 1990 while other regions like the East Asia and the Pacific regions have seen on average an annual decline of 4.5%. It is expected that a 5.4% decline per annum is required to achieve the millennium development targets in the sub-Saharan African region (Wagstaff et al., 2004). This is obviously a tall mountain to climb for countries in this region.

Among the knowledge base on why maternal deaths occur and how to avert them, access to maternal health services is a primary intervention for achieving better maternal health outcomes (Maine et al., 1999; Thaddeus et al., 1994; Bour, 2003). Notwithstanding this, the organization of maternal service and how maternal health service is financed have also been seen to play a part in the health-seeking behavior in general and outcomes (Oppong et al., 1994; Agyepong, 1999; Witter et al., 2007; Witter et al., 2008).

It may seem right to discuss maternal health outcomes based on only how maternal health service is organized or on how it is financed. But such discussions may lead to a one-sided finding, if not biased outcomes. Therefore, the decisions and choices of maternal services influenced by the organization and recent health financing reforms are the primary focus of this study. It is against this backdrop that the paper attempts to: 1. determine the choice and use of maternal health facilities; and 2. examine the influence of organization and financing of maternal health services on attitudes to seeking maternal care.

This paper presents outcomes on maternal health-seeking behavior after the country had introduced the delivery exemption policy alongside the organization of maternal health services. However, there have been few studies that have examined the impact of this policy on health-seeking behavior (Deganus et al., 2006; Asante et al., 2007; Penfold et al., 2007). Studies that have been carried out after the policy have either looked at the financing part in isolation or the cost-effectiveness of the intervention (Arhinful et al., 2006; Deganus et al., 2006; Witter et al., 2007). There are also other researches that have shown results on the effects of the reform on maternal births and incomes of health workers (Bosu et al., 2007; Witter et al., 2007). This paper therefore, contributes to the extant art of knowledge by examining maternal health-seeking behavior from a health system perspective that combines the organization and financing of maternal health services.

The remainder of the paper is structured as follows: section 2 discusses the health system, beginning with human resource and organization of maternal health services. The financing of maternal health services at the national and district levels are also discussed in this section. The methods used in the study are presented in section 3. The case study results, which highlight the influence of health system on maternal health-seeking behavior, are examined in section 4. Finally, section 5 deals with the conclusions and recommendations of the paper.

### 2. The Health System in Ghana

Since maternal health care relies on the entire health system of a country, its outcomes including health-seeking behavior can be traced from the way health systems operate (Parkhurst et al., 2005). The health system includes the human resources, organization of maternal health services which dwells on the availability of both private and public sectors, and reforms in the health sector (Graham, 2002). These are in general, what Ghana's maternal health service is dependent on, and have various implications for maternal health-seeking behavior. This section of the paper discusses these elements in the health system.

Firstly, human resources in maternal health service are generally understood as the presence of skilled birth attendant during delivery (Parkhurst et al., 2005; Hoope-Bender et al., 2006). This can be a doctor, midwife, nurse and the increasing numbers of traditional birth attendants (TBA), who may not have any midwifery training (WHO, 2004). It is the accessibility to these skilled birth attendants that affects the maternal health outcomes. However, studies have shown that the presence of these birth attendants may be meaningless unless they are well coordinated so that they become accessible, and also resourced enough to do what is expected of them should complications or in the extreme case deaths occur (Graham et al., 2001; WHO, 2004; Parkhurst et al., 2005).

Secondly, both the private and public health services influence delivery of maternal health services and maternal health outcomes (Parkhurst et al., 2005). Accordingly, people tend to seek health services in private or public health facilities based on, perhaps their ability and willingness to pay. However, people's ability and willingness to pay for health services are subject to their incomes. The differences in income tend to create disparities and inequalities in health-seeking behavior thus affecting maternal health outcomes (Oppong et al., 1994; Agyepong, 1999). The disparities are wider in developing and transition economies (WHO, 2005), of which Ghana is no exception. We can argue that, if income can determine the facility where women seek for health care, then the quality of healthcare availability can be established. This stems from an assertion that, private health facilities are inclined to offer quality services as compared to public health facilities (Wilson et al., 1997; Graham et al., 2001; Parkhurst et al., 2005). The low quality of healthcare in the public health facilities is in consequence of the exodus of well-qualified human resources from public health facilities to their private counterparts as a result of attractive incentive packages the private health sector offers them (i.e workers).

Thirdly, reforms pertaining to health care are happening globally. Particularly, in sub-Saharan Africa, some of the key reforms are decentralization of health services and the sector-wide approach (SWAp) (Peter et al., 1998; Mayhew, 2003). In Ghana for instance, there have been models of decentralizing the public administrations with defined functions at the national, regional and district agencies. However, the role of the central administrations still remains stronger (GOG, 1996; Mayhew, 2003). The Ministry of Health (MOH) retains policy-making functions while the regional hospitals and district health management teams have the status of Budget Management Centers (GOG, 1996; Agyepong, 1999; Mayhew, 2003). The exact roles and functions of these decentralized offices remain overlapping and mixed in many instances (Mayhew, 2003). The control from the centre also affects funds flows, since the MOH assumes the responsibility of staff recruitment and payment, as well as budgetary allocations and planning specifications (Agyepong, 1999; Mayhew, 2003). Consequently, the district level may not have authority over who is hired or the human capital at their disposal. These mixed functions within the system affect health programs -such as antenatal care and delivery- which are pertinent for maternal health outcomes.

The SWAp was introduced in most developing and transition countries to de-concentrate funds and streamline them to certain assigned projects that are prioritized with budget ceiling at the national level (Cassels et al., 1998; Hutton et al., 2004). In Ghana, the SWAp was introduced to increase coordination of funds since its health sector relies heavily on donor support (Goodburn et al., 2001). Even though this practice in some cases has led to the delay of funding to the district level from the government (Mayhew, 2003), what is worth considering is how reforms can shape maternal health services and eventually, maternal health outcomes.

In conclusion, these reforms address: how health sector funds are distributed; the merging of separate health services or privatization of services; and the re-organization of health care delivery (Parkhurst et al., 2005). So far, the reforms that have been undertaken in Ghana have affected the local systems, changed the nature of incentives for health workers, and regulated and improved accountability at all levels of the health sector (Agyepong, 1999; Mayhew, 2003).

### 2.1 Financing Maternal Health Care in Ghana

As at 2010, the maternal mortality ratio in Ghana stood at 350 deaths per 100,000 live births, making it one of the countries with high rate of maternal mortality (Population Census, 2010). In order to reduce maternal mortality and meet millennium development targets by 2015, there was the need to clear some barriers that hinder women in seeking maternal health care. Some of the targeted barriers include financial challenges, which is one of the teething issues that obviate women from seeking maternal health care. Accordingly, the provision of free maternal care under the delivery exemption policy was introduced in 2004. This policy was financed by the local government ministry through the Highly Indebted Poor Countries (HIPC) debt relief fund (Witter et al., 2009).

The exemptions for delivery care program began earlier than the National Health Insurance Scheme (NHIS), but later on it had to be financed through the NHIS in 2008 and other means (MOH, 2004; NHIA, 2008). The policy exempted pregnant women both insured and uninsured under the NHIS from paying facility user fees during pregnancy check-ups and delivery. Although this policy did not reduce facility cost^1^ to zero, it granted pregnant women access to virtually free antenatal, deliveries and postnatal care in many health facilities (Armar-Klemesu 2006; Witter et al., 2009). The additional merit of the delivery exemption policy was related to financial barriers, which were removed particularly for poorer women. However, non-facility cost (such as transportation cost) was not included in the policy.

The NHIS programme is centrally administered and it is funded through formal and informal sources of funding. The deductions from Social Security and National Insurance Trust (SSNIT) and government budget allocations are formal contributions to the National Health Insurance Fund (NHIF). Annual premiums that range between 3.6 USD and 24 USD per head based on income and ability to pay form part of the informal contributions to the fund (NHIA, 2008; NHIA, 2010). Also, taxes (both direct and indirect) that are levied^2^ on selected goods and services go into the fund as informal contribution (GOG, 2003). These contributions are supported with grants, donations, and gifts.

The National Health Insurance Levy (NHIL) from taxes accounted for about 61% of total income of the NHIS in 2009 (NHIA, 2010). Formal sector contributions made up 15.6%, while the informal sector premium was only 3.8% the same year. The NHIF provides funds for the scheme and financially support people who are not able to pay. The scheme is designed to promote social health protection through risk equalization, cross subsidization, solidarity, equity and quality care. The NHIS also reduces unexpected expenditure on health care and catastrophic spending among the insured. The scheme also exempts certain category of individuals from paying annual premiums such as children under 18 years and adults above 70 years (NHIA, 2008; MOH, 2009).

Notwithstanding the National Health Insurance reimbursements, other means by which the regional and district level health services can source finances include; internally generated funds, funds from Non-Governmental Organizations (NGOs), Government funds and minimum contributions of cash and in kind from philanthropists (Agyepong, 1999).

### 2.2 Health Services in Bosomtwe District

There are three sub-districts and 63 communities in Bosomtwe district. The estimated population is 93,910 as at 2010 (Population census, 2010). Health service supply is organized in 14 Community Health Planning Service (CHPS) zones (GHS, 2009). There is staff strength of three hundred and eighty-eight (388) health personnel (public and mission). Out of this, one hundred and eighty-four (184) work for private and mission health facilities and 118 are Ghana Health Service (GHS) personnel in the public health services. Table 1 gives a description of the type of health professionals working in the district.

**Table 1:** Distribution of different types of health personnel

Type	Number of staff	Percent (%)
GHS	92	30.42%
CHAG/MISSION	184	47.43%
CASUALS	14	5.42%
HEW	62	12.63%
Attachment	10	2.58%
Service personnel	6	1.55%
Total	388	100%

Also, health service delivery is carried out in sixteen (16) public and private health institutions. These institutions are made up of four (4) government facilities, seven (7) CHAG or Mission and five (5) private facilities. The district also has 38 outreach points that offer Reproductive and Child Health Services (GHS, 2009). There is also community based surveillance program in the district, which employs volunteers who have the responsibility to record and report diseases, deliveries and deaths in their various communities on monthly basis.

There are other non-orthodox treatment centres in the district. Table 2 shows the types of health facilities both public and privately administered hospitals, clinics and maternity homes in the district. Currently, there is collaboration between the health directorate and other health centres in the district to enhance health service delivery.

**Table 2:** Types of health facilities in the district

Hospitals	Health Centres	Clinics	Maternity Homes
Kuntenase *(G)* Hospital *(G)*	Jachie H/C *(G)*	Nyameani C. *(M)*	Sophia Mat. *(P)*
St. Michael's *(M)* (Hospital *(M)*	TetrefuH/C *(G)*	Brodekwano C. (*M)*	Comfap Mat. *(P)*
Divine Mercy*(P)*	Piase CHPS *(G)*	Amakom Clinic *(M)*	Emmanuel Mat. *(P)*
		Feyiase Clinic *(P)*	
		SDA Clinic *(M)*	
		St. Mary clinic *(M)*	

NB: G – Government Institutions, M – Mission, P – Private

Furthermore, the sources of funding to the district health directorate come from the donor pool fund, internally generated fund and government subvention (GHS, 2009). The internally generated fund includes funds from the NHIS and fees paid by patients, which are not covered by health insurance. The Ministry of health funds are also part of their sources of fund to the district health directorate.

NB: G – Government Institutions, M – Mission, P – Private

However, this fund is usually earmarked for specific programs (for instance, TB Care or malaria program). The district health directorate can also be supported by the district assembly with funds from the district assembly common fund when the need arises. The district assembly usually helps the health service directorate with donations and in kind. Holding this picture of the health system of this district in our minds, we will present the interviews and group discussions to examine what impacts such resources (personnel), organization and financing of district health service can have on maternal health-seeking behavior in section 4 of this paper.

## 3. Methods

Yin (2011) posits that qualitative research approach has currently become an attractive-if not the mainstream-sort of research in both academic and professional operations. Since this study wanted to access and produces an in-depth and adequate data essential for the study's analysis, a qualitative research approach was employed. Also, in order for the researchers to thoroughly evaluate and examine the data that were collected from the women in their natural setting, a case study design was applied to complement the qualitative research method.

### 3.1 Case Selection

The diverse case method-a non-random purposive procedure-was used to select the cases. This method has the capability to handle differing cases within categories and also explain the outcome through the different cases. The method covers all the relevant range of variation in cases, which enhances the representativeness of the variability in the population (Gerring, 2008). Women were selected based on indicatives such as delivery at home, at health facility and women with peculiar experiences before, during and after birth. However, for a woman to be included in this study, she must have given birth not more than a year prior to data collection. This allowed us to minimize recall bias in the responses of the informants. Other key informants^3^ including a doctor, three midwives and a trained traditional birth attendant were also selected. Table 3 below depicts the number of informants by age and the health zones. The distance (km) of the health zones from the district hospital are also shown.

**Table 3:** Informants by age and residence related to distance from hospital

Age group (years)	Kuntenase zone (0-5km from hospital)	Abono zone (28-50km from hospital)	Total
15-25	3	1	4
26-37	4	5	9
≥ 38	1	2	3
Total	8	8	16

### 3.2 Data Collection Techniques

The study used interviews with a semi-structured guide to collect data from informants (Yin, 2009). The advantage of flexibility in semi-structured guide to changes in the order and form of questions, such that every informant can be probed when interesting and peculiar issues to an individual are encountered was crucial for our choice of this type of guide (Crang et al., 2007; Kvale et al., 2009). The questions for the interviews were prepared with relevant areas regarding factors that influence women decisions and choices of maternal health services.

Two group discussions were also conducted, one in each of the selected towns for the study. In order to make a comparative analysis, the selection of the women in Kuntenase and Abono was based on travelling distance to the district hospital, which created a natural disparity between women living in these two towns. These discussion groups were made up of fairly homogeneous women. We made sure that, the women who took part in the group discussions had already been interviewed one-on-one during the interview phase.

The questions for primary informants were prepared in sections. Each section had sets of questions to decisions and choices of maternal health services. The questions that centered on decisions on maternal health services were; decision on facility to use for delivery and factors that influenced the decisions-for instance social relations (close relatives, friends, or hospital staff they know). Other questions were also centered on choices of facility for maternal care (for instance, antenatal care and place of delivery) and the type of assistance received during delivery. Questions on knowledge and need for maternal care and barriers to maternal health care were also asked. Their knowledge of Traditional Birth Attendants (TBAs) or other health care providers (midwife) in the communities nearest to them were asked. Their knowledge on rights to certain type of services in the hospital and patient satisfaction were included in the questions. There was also a section on the background information of our informants that centred on age, marital status, level of education, occupation and residence.

Informant consent was sought from all participants. Informants were also aware if their direct quotes were used in the reporting. All the interviews and discussion groups were moderated by the researchers. The questioning and answering occurred in a calm and serene atmosphere that enabled the researchers to tape-record the responses with small tape recorder. Following Ravasi and Zattoni (2006), the various transcriptions were supported with contact summary sheets and interview notes. Both the interviews and focus group discussions were held in local Akan language, but during the reporting stage, all the quotations were translated into English.

### 3.3 Text Analysis

Texts that were transcribed from interviews and group discussions with the women were analysed using selective coding method. Selective in the sense that those interesting, diverse and rival comments in the conversation were brought under related broad themes formulated from the research objectives. The content of such comments from the transcribed interviews were studied to check for patterns and how they related to the concepts and analytical approaches used.

### 3.4 Limitations

Although the small sample selection reduces the strength of generalization, in order to make it possible, a replication method was applied in this study. Yin (2009) argues that replication logic is the same logic that underpins each case and be applied in all cases. The study employed a common technique for data gathering and this method was directed by a case study protocol. This design was chosen to strengthen external validity of outcomes and make the results closely representative of the population (Gerring, 2008; Yin, 2009).

## 4. Results

All the examples on health-seeking behavior among the women happened after the introduction of the delivery exemption policy with major shifts in the financing of maternal health from local government to national insurance coverage.

### 4.1 Study Participants

From the interviews, important background information of informants concerning age, level of education, occupation, and parity were analysed. The average age for the women involved in the study was 26 years. The oldest among was 39 years and the youngest, 21 years. The ages of informants also reflected in the number of children they had. On average, the number of children (parity) an informant had ranged between 1 and 7 children.

All our informants had formal education with the least having completed primary education. The highest level of education our informants had was high school and there were only two of such among our informants. The type of occupation informants were typically engaged in was farming and trading. It was only one of our informants who was unemployed. From our analysis of the interviews with the women, we tried to observe whether there were any differences in maternal health seeking behavior and background information of women. We did not observe any consistent difference between women in their health seeking behavior and type of occupation they were engaged in. However, differences were observed for age, parity and level of education, and the use of maternal health services. For instance, those who had higher levels of education tend to use higher levels of care (i.e. hospitals) than women with lower levels of education who usually give birth at home or with a traditional birth attendant. Also, women who had experienced more than one birth, especially beyond four children, tend to give birth at home while those experiencing their first delivery utilize the health facilities.

### 4.2 Health System and Health-Seeking Behavior

The observable facts of the study highlight that the various characteristics of maternal health system are considered as determining factors on women's behavior and decision to seek care, choice and use of maternal health services. These characteristics include location of health facility, order of referrals, capacity of health facility, and also how the financing of maternal health care through the delivery exemption policy influences health-seeking behavior. These aforementioned characteristics of the maternal health system are examined in subsequent sub-sections.

### 4.3 Who Pays and Behavior Outcomes

Some enabling factors brought about by structural policies influence the health seeking behavior of the women in Bosomtwe district. The introduction of virtually free maternal health services under the delivery exemption policy financed under the NHIS has created new forms of health seeking behavior among women. However, there are marked differences for seeking maternal health care from conception to delivery. For instance, the attendance of Antenatal clinic (ANC) has increased tremendously. Women in the study are more aware of the need to seek maternal care during pregnancy to know their health status. Even though distance is still a barrier, the use of health facility for antenatal check-up has increased and women tend to use it more than they actually need to. This is termed as ‘*moral hazard*’ (Philip 1990). As a result of the insurance coverage, all the women we interviewed attended ANC at least more than twice before delivery. The situation is quite different with deliveries as labor can suddenly happen. For instance, labor may occur in the night and there will be the need for delivery at home if there are no easy forms of transport for the women, especially those from locations far from the facility.

### 4.4 Capacity of Facility and Behavior Outcomes

The position of a health facility in the hierarchy of health structure provides an idea of how the facility is resourced and the kind of services it can provide (Bergstrom, 1994; Ojeifo, 2008). For instance; the capacity of health facility can be in terms of number of staff (both skilled and partially skilled), number of delivery wards, obstetric logistics and the level of technology.

In the Bosomtwe district, the health facility which is well resourced is the district hospital. The district hospital is supported by other lower level care providers such as the clinics and other formalized maternal care services like trained TBAs that provide maternal health services. Interviews with key informants reveal that both the district and other supporting lower facilities are under resourced in terms of staff, equipment, and technology to provide satisfactory services. In a group interview with midwives on their resources, some mentioned that;

‘‘The hospital has three midwives. There is only one labor ward here… and only one delivery pack, which needs ‘first class’ disinfection for so many hours before it can be used for other deliveries. The women are therefore, told to bring items such as carbolic soap, disinfectants, white or grey cloth for this exercise.’’

The clinic and trained TBAs in the communities also have virtually no resources at all. An interview with a trained TBA who has been practicing for over 8 years and claim to have assisted in uncountable deliveries had this to say;

‘‘[All] I have is my blade, tread, hand glove and a cloth, [which are] necessary to assist women during delivery. After the assistance, I take only some few gifts and items from the women to bless my soul. This is because the work is difficult.’’

This description shows how resourced auxiliary health providers like the trained TBAs are and the level of assistance they can provide, should complications occur in the remote communities of the district. These trained TBAs serve as first point of call for most of the women in the rural areas when they are in labor and require immediate assistance. However, in most instances, hygiene surrounding deliveries are compromised, which can possibly increase the risk of postpartum infections (Ojeifo, 2008).

The hospital staff has instituted a form of facility use cost through a collection of items such as disinfectants, soaps and white *Kaliko* cloth to supplement their materials. There are therefore, strong tendencies that the choice of place for delivery will be at home without supervision since women who cannot afford such items will be inclined to deliver at home though they still use the hospital for antenatal care.

### 4.5 Decision to Seek Maternal Health Care

The decision to seek maternal care can be earlier, delayed and spontaneous. This depends on the situation that prevails in the whole period from conception to delivery (Bergstrom, 1994). The decision made when choosing a health facility for maternal health services, whether delayed or spontaneous, may come from the woman, some close relatives and where the woman resides. It is evident that decisions to choose a maternal health facility lie with the help of ‘significant others’ who are either the mother or grandmother of the woman. For instance one of the women interviewed said: *“I am staying with my mother and she said it would be proper if I gave birth at the hospital”*. For another woman it was mostly common for her to say *“it was my mother's advice for me to give birth at home”*.

This is usually connected to women with cases of first birth experience and their proximity to the hospital (Bour, 2002). Women with high parity have more autonomy in deciding for themselves where to give birth. Close relatives with high parity and extensive experience with birth are also highly regarded in circumstances pertaining to decisions and choices of health facility. This is connected to cultural reasons where the mother or grandmother helps in taking care of the baby and their ability to assist in delivery.

Informants from Kuntenase health zone have more autonomy to decide the type of health facility for birth. This is due to their proximity to health facility and their socioeconomic status like education and income levels. Their locational and effective access opportunities are high and thus influence their health-seeking behavior. Women are able to make a decision on where to give birth based on their knowledge on the use of the facility in their first experience or from friends who had experienced it (Gay et al., 2003). Informants who choose home for births do so on the basis of their past experiences in regard to home births. For instance, a 33 year old informant with seven children said:

*“I have never given birth in hospital before. When I take orthodox medicine, I fall sick. In the beginning I had no knowledge with births and my mother assisted me always, but from my fourth child onwards I had given birth alone unassisted*.*”*

The influence of social relation on decision of women and health seeking behavior applies not only to women farther away from health facilities, but also women who are closer to a maternal health facility. Women with lower parity have their close relative deciding in almost all cases and the appropriate time for them to go to the facility when in labor. On the contrary, for periods when user fees for maternal services applied, women with low economic status virtually depended on their husbands. With the introduction of the free delivery exemption policy, women are free to decide which facility to use for antenatal care and birth. This is an example of norm and value changes resulting from financing reforms in the health sector (Valtonen, 1994; Mikkelsen, 2005).

### 4.6 Reasons for Delivery at Home

Delivering a baby at home particularly, without professional supervision can be risky and may come with unforeseeable consequences (Okafor et al., 1994). From the perspective of our informants, reasons for giving birth at home were discussed in groups, especially for those who had distance disadvantage. There are some home births that are supervised by trained traditional birth attendants and others without any supervision.

In the Abono community health zones, which are served by lower order facilities, home birth is a common practice. Some women make preparation for home birth and give birth at home. This is due to their knowledge about the hospital resources and other reasons. Accordingly, two women had the following to say:

“For children that walk in the sand, I have ten of them and three have passed away. I gave birth to nine at home and the last in the hospital for which I suffered complications. Home birth is just like the hospital. In the hospital, no one will give birth for you. All they do is to help in some cases with injection and water drips (am I lying?). Otherwise, everyone will give birth at home.”

“I have seven children and I gave birth to all of them at home. From the fourth child onwards I always gave birth at home unassisted. I always give birth at home because when I take orthodox medicine I become so weak. I usually use herbs from my village before and after birth. I have never given birth at the hospital but I am always strong by God's grace.”

The perception of women in the Abono health zone is that sometimes hospital care conflicts with the use of traditional forms of care. However, for those who had experienced hospital care, the use of hospital is not different from home birth and they can substitute hospital birth for home birth if they are sure of uncomplicated birth, based on their antenatal care examinations.

Among women with first birth experience and those that expected complicated births, the need for specialised maternal care is considerably intensified. Women with knowledge of signs of complicated birth, pregnancy related illness and first birth experiences prefer the hospital to home as place of giving birth. In the Kuntenase community health zone, hospital birth is common and women who give birth at home are looked upon strangely. From the interviews, it is clear that giving birth at home is not all about experience with birth, but also other factors such as insurance coverage and distance from the health facility.

*“It was in the olden days that women gave birth at home. Nowadays no one should tell you to go to the hospital more especially, when you do not have to pay anything. When you look at the level of civilization, you follow the world as it moves and knowledge is increasing*.”

This was the view of a 32 year mother with 2 children and secondary level education.

The risk of giving birth at home is considered high for women in the Abono health zone. The risk is however, perceived to be lower for women in communities in the Kuntenase health zone. This behavioural margin among the women in these two health zones is partly as a result of the distance in reaching the nearest health facility for birth, what forms of knowledge they are predisposed to, health beliefs and the level of risk the women can allow (Philips, 1990; Olujimi, 2007). The access opportunities of women and structure of the health care delivery system in terms of levels of care strongly influence the behavior of the residents in a particular area. For instance, all other factors remaining unchanged, once a woman in the Abono health zone is successful with her first home birth, the rest of the children may be born at home unless there are serious complications that will require critical referral. Women in the Abono health zone do not feel being treated unfairly with provision of health services if complications are minimal with the kind of services they receive. Thus an important finding is that, inequality does not necessarily imply inequity for women in the Abono health zone if complications do not occur.

### 4.7 By-passing Health Facility

The concept of ‘by-passing’ health facilities is the situation where for instance, a patient uses a higher order facility for a treatment that a lower order facility can offer (Oppong et al., 1994). This situation can occur due to the resources of a particular facility, organization of health service delivery and the nature of health care financing.

For instance, the empirical facts highlight that pregnant women misperceive the use of levels of maternal health services both higher and lower. Usually antenatal care is sought from a higher order facility like the regional hospital. From the perspective of a midwife who works in the district hospital, ‘bypassing’ occurs because;

“Some of the pregnant women are petty traders who trade in all kinds of goods and services in the city. They sell in the city all day and come back to their villages in the evening. They think the nearest facility for antenatal care is the regional hospital. As such we [hospital staff] do not have any records on them [women]. Some therefore, either deliver at the regional hospital or at home.”

Women, who do not have records in a particular health facility, do not prefer giving birth in that facility since they are not sure of receiving satisfactory services. We observed that bypassing of facilities are not only due to economic activities women engage in, but also other social outcomes such as finding out which one will provide better services (Oppong, 1994). For instance, some of our informants visit more than one maternal health facility for antenatal care. This is what a 28 year mother with first birth experience in an interview had to say:

“… some women say if you are pregnant you should visit at least two health facilities so that during labor you choose the one which you think you received enough care and satisfaction of services from the hospital staff.”

Consistent with the study of Okafor et al. (1994), women now have more options in services and visit many facilities as basis for reference and comparison because, they are exempted from fees irrespective of the facility they use, if only the facility is registered with the NHIS to give ‘free’ maternal health care. These ‘rational’ decisions with regards to visiting more than one health facility lead to misuse of levels of health services. The connection between the organization and financing of health services and how the health system shapes health-seeking behavior is clearly demonstrated here.

### 4.8 Barriers and Behavior Outcomes

Access to maternal health care may be interspersed by inability to use the health services provided by health facilities. The users of maternal health services may be discouraged from using the services delivered (Valtonen, 1994). Some of the considerations that may limit the use of services from a particular health care facility may be manifold including; geographical, medical, socio-cultural and knowledge barrier results from health system, and human orientation (Thaddeus et al., 1994; Barnes-Josiah, 1998).

The geographical barrier which has to do with the distance between the service providers and recipients is more entrenched for women farther away from a health facility. In some remote towns, the unavailability of transportation and the cost of transportation are severe all year round and thus sometimes the use of boats that are mostly serving tourist purposes on the lake Bosomtwe are used to transport women in Labor. This points to transport barriers to maternal care, which causes delay in reaching the facility. Here is a descriptive example of an extreme geographic barrier one woman told us during the group discussion.

‘‘… [T]he women who live on the other side of the lake have even worse situation. Sometimes they have to cross the lake before they get to the nearest hospital to deliver. At first it was forbidden to cross the lake when pregnant, but now we cross and it is even easier with some bridges at some places.’’

### 4.9 Medical Barriers and Behavior Outcomes

The structure and organization of maternal health services present medical barriers for women seeking care. The difficulty with referral of patients to another level of care, waiting time at the hospital, and other partial user fees at some health care levels raise pressing issues for consideration.

The waiting time at the hospital at the hospital a stage of delay in using health services (Maine, 1993; Barkat et al., 1995). In the district hospital, which has one labor ward, waiting times are longer even after arriving at the facility. The longer waiting periods are as a result of the capacities of the hospital in terms of logistics and materials to deliver satisfactory services. This situation makes women wait at home until they feel that their time is fully due and this may increase the risk for giving birth at home unassisted. This is consistent with the findings of Wilson et al. (1997).

Another delay related to women in labor, having to wait at one level of health care is the difficulty with referral from one level of care to another. The lower order levels of care for instance, trained TBAs; believe they are capable of assisting delivery in all conditions. Critical accessibilities from a trained TBA to midwife in the hospital are sometimes delayed because, trained traditional birth attendants are not resourced enough to refer and accompany pregnant women, hence ensuring continuity of care.

A typical medical barrier observed was partial user fees and bottlenecks introduced by hospital staff to supplement the capacity of facility. Even though maternal health services are virtually free as a result of the NHIS, lower order maternal health care services like the trained TBAs who are not enrolled on the NHIS accept gifts from women as fees. Also, the hospitals, clinics and trained TBAs are under resourced and therefore, collect items such as carbolic soaps, disinfectants and *Kaliko* cloth to assist delivery. These items are collectively known as ‘dropping prices’, which are the new user fees in a sense that, they have come to replace the user fee and increased cost burden for some women whose social statuses are low.

## 5. Conclusion

**Figure 1:**
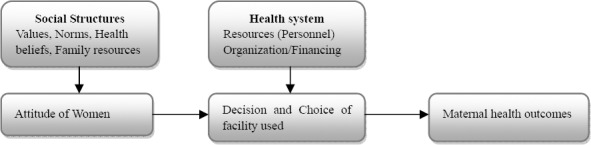
Influential health-seeking behavior elements in the health system and facility used

This paper highlights the sort of ramifications the health system in general can have on maternal health-seeking behavior and eventually, maternal health outcomes. The study reveals that the health systems (such as human resources and organization/financing) serve as barriers and filters between the women, and the actual facility women decide on and choose for antenatal and delivery. Interestingly, the study also illuminates certain social structures (such as values, norms, health beliefs and family resources), which health planners are to pay a particular attention to. These social structures influence the decisions and choices of women in selecting the kind of health facility for their antenatal and delivery. Figure 1 below recapitulates the findings of the study.

Source: Authors’ own for purpose of this research

We recommend that since the decision and choice of women are influenced by the social structures and the health system, it is vitally important that discussions on maternal health planning and policy ought to encompass the entire health system and the social structures in order to achieve the needed maternal health outcomes. Recent debates and discussions on maternal health outcomes in sub-Saharan Africa must address the entire health system as well as the social structures. Even though modest progress in maternal health outcomes has been achieved in the last decade, a closer look of these perspectives on maternal care can help improve maternal health outcomes.

Also, women should have the right to choose to give birth at home. Meanwhile, the risk associated with such deliveries can be minimised if trained traditional birth attendants are somehow supported and financed at the district levels. In addition, indirect health care cost such as transportation and certain petty charges at the facility should be brought to the barest minimum. These changes will go a long way to shape not only maternal health-seeking behavior, but also maternal health outcomes.

## Footnotes

1 Facility cost refers to costs related to use of facility eg. costs of normal deliveries or caesarean sections

2 This is termed as National Health Insurance Levy (NHIL)

3 Key informants are people with special knowledge and ideas about a phenomena or topic (Mikkelsen, 2005)
